# Recovery cycles of posterior root-muscle reflexes evoked by transcutaneous spinal cord stimulation and of the H reflex in individuals with intact and injured spinal cord

**DOI:** 10.1371/journal.pone.0227057

**Published:** 2019-12-26

**Authors:** Ursula S. Hofstoetter, Brigitta Freundl, Heinrich Binder, Karen Minassian

**Affiliations:** 1 Center for Medical Physics and Biomedical Engineering, Medical University Vienna, Vienna, Austria; 2 Neurological Center, Maria Theresien Schloessel, Otto Wagner Hospital, Vienna, Austria; University of Pittsburgh, UNITED STATES

## Abstract

Posterior root-muscle (PRM) reflexes are short-latency spinal reflexes evoked by epidural or transcutaneous spinal cord stimulation (SCS) in clinical and physiological studies. PRM reflexes share key physiological characteristics with the H reflex elicited by electrical stimulation of large-diameter muscle spindle afferents in the tibial nerve. Here, we compared the H reflex and the PRM reflex of soleus in response to transcutaneous stimulation by studying their recovery cycles in ten neurologically intact volunteers and ten individuals with traumatic, chronic spinal cord injury (SCI). The recovery cycles of the reflexes, i.e., the time course of their excitability changes, were assessed by paired pulses with conditioning-test intervals of 20–5000 ms. Between the subject groups, no statistical difference was found for the recovery cycles of the H reflexes, yet those of the PRM reflexes differed significantly, with a striking suppression in the intact group. When comparing the reflex types, they did not differ in the SCI group, while the PRM reflexes were more strongly depressed in the intact group for durations characteristic for presynaptic inhibition. These differences may arise from the concomitant stimulation of several posterior roots containing afferent fibers of various lower extremity nerves by transcutaneous SCS, producing multi-source heteronymous presynaptic inhibition, and the collective dysfunction of inhibitory mechanisms after SCI contributing to spasticity. PRM-reflex recovery cycles additionally obtained for bilateral rectus femoris, biceps femoris, tibialis anterior, and soleus all demonstrated a stronger suppression in the intact group. Within both subject groups, the thigh muscles showed a stronger recovery than the lower leg muscles, which may reflect a characteristic difference in motor control of diverse muscles. Based on the substantial difference between intact and SCI individuals, PRM-reflex depression tested with paired pulses could become a sensitive measure for spasticity and motor recovery.

## Introduction

Posterior root-muscle (PRM) reflexes [1,2] are short-latency spinal reflexes evoked by epidural spinal cord stimulation (SCS) or by skin surface electrodes placed in a configuration to generate a current flow that partially crosses the thecal sac (transcutaneous SCS) [3,4]. They result from the stimulation of proprioceptive fibers within posterior rootlets/roots, which reflexly activate motoneurons in the spinal cord [5–8], are electromyographically recorded as reflex compound muscle action potentials, and can be obtained in practically all lower extremity muscles by a single stimulation pulse with an intensity exceeding the excitation thresholds of the respective lumbar and upper sacral posterior roots [9,10].

In all contemporary studies demonstrating unprecedented locomotor recovery in individuals with severe spinal cord injury (SCI) by epidural SCS, PRM reflexes were employed to intraoperatively guide electrode placement overlying the L1–S2 spinal cord segments [11–14], assuming that they are generated through direct, segmental projections of the stimulated afferents to the homonymous motoneuron pools [2,5,15]. Further, tonic and rhythmic muscle activity in paralyzed legs induced by epidural SCS are largely composed of series of PRM reflexes [1,6,16]. Similarly, in studies examining the potential use of transcutaneous SCS for spasticity and motor recovery, PRM reflexes were evoked to confirm the position of the stimulating electrode over the lumbar spinal cord [17–21]. In addition, transcutaneously elicited PRM reflexes are being used in physiological studies to extend the information gained from the classical soleus-H reflex to many muscles simultaneously [22–26]. Indeed, they share key characteristics with the essentially monosynaptic H reflex evoked by electrical stimulation of group Ia muscle spindle afferents within the mixed tibial nerve [27–32]. Like the H reflex, the PRM reflexes are suppressed by tendon vibration [2,33] and are modulated by passive or active leg movements in a task specific manner [2,9,22,34].

A hallmark behavior of the H reflex is described by its recovery cycle, i.e., its depression and gradual recovery when tested by paired pulses [35–39]. Specifically, the recovery cycle is the time course of amplitude changes of a test-H reflex elicited at progressively increasing intervals after a conditioning H reflex. In neurologically intact individuals, the H-reflex recovery cycle is characterized by a complete suppression for the first tens of milliseconds, an initial peak of incomplete recovery around 250 ms, and slow recovery to the size of the unconditioned control response at 10–15 s [37,40]. Remarkably, results of two previous studies in individuals with intact nervous system suggested that PRM reflexes may have a different recovery cycle: An early exploratory study found a slower recovery of triceps surae-PRM reflexes and assumed that this followed from the concomitant activity in the PRM reflex arcs of other muscles [41]. Another study found a slower recovery of the soleus-PRM reflex than of the H reflex when tested at intervals of 25–200 ms, but only during background voluntary contraction [42].

Further, the leading mechanisms underlying the H-reflex recovery cycle, presynaptic inhibition [43,44] and homosynaptic depression [45,46], are reduced after chronic SCI [47–49] and contribute to spasticity [50,51]. In line with these pathological changes, a study suggested that the depression of the H reflex with decreasing interstimulus interval from 10 s to 1 s was reduced in participants with SCI [52].

Here, we first evaluated the recovery cycles of the H reflex and the PRM reflex of soleus in individuals with SCI as well as uninjured controls for paired pulses with interstimulus intervals of 20–5000 ms. We hypothesized that both H-reflex and PRM-reflex recovery cycles would differ significantly between neurologically intact and SCI subject groups and that the difference would be greater for the PRM reflex. We further hypothesized that the recovery cycles of the H reflex and the PRM reflex would differ significantly with respect to within-subject group comparisons. We assumed that these differences would result from the multi-root input associated with transcutaneous SCS, the heteronymous connections in the human spinal cord [48,53,54] and their repeated activation [55,56]. The depression of the PRM reflex would thus depend on a wider range of spinal inhibitory mechanisms, which are altered after SCI. Further, we obtained for the first time PRM-reflex recovery cycles of rectus femoris (RF), biceps femoris (BF), and tibialis anterior (TA) in addition to that of soleus, which allowed us to identify potential differences between spinal reflex regulation of diverse muscles.

## Materials and methods

### Subjects

Data were derived from ten neurologically intact volunteers (four males, mean age ± SD: 28.6 ± 5.1 years, ranging from 22–36 years) and ten individuals with traumatic, chronic SCI (mean age: 40.1 ± 18.0 years, ranging from 18–66 years; no age difference between subject groups, Student’s t-test, P = .067). Details on the neurological status of the individuals with SCI evaluated according to the International Standards for Neurological Classification of Spinal Cord Injury [57] are provided in Table 1. All individuals with SCI had lower-limb spasticity, clinically evaluated based on the Modified Ashworth Scale (MAS) [58]. To obtain a single, comprehensive measure for lower-limb spasticity, the individual MAS scores from twelve separate tests of both legs were summed up (with a value of 1.5 for the 1+ scoring category) to result in a MAS sum score ranging from 0–96 (0, no increase in muscle tone). The movements tested were flexion, extension, abduction, adduction, as well as internal and external rotation of the hip; flexion and extension of the knee with the hip in an extended position; and ankle dorsiflexion with the hip and knee in a flexed position as well as dorsiflexion, plantar flexion, and pronation of the ankle with the hip and knee in an extended position. Subjects 2, 4, 5, 8 and 10 were on oral antispasticity medication (Table 1) and had taken the last dose 12–15 hours prior to their participation. The elimination half-lives of tizanidine and baclofen are relatively short, amounting to about 1–3 hours and 3–6 hours, respectively [59–63]. The elimination half-life of tetrazepam, taken by two participants, is 15–22 hours [64,65], yet effects on H-reflex excitability measured two hours after administration were shown to be marginal or not significant [66]. None of the participants used drugs to treat depression, anxiety, or attention deficit disorder. Among the exclusion criteria were active and passive implants at vertebral level T9 or caudally, such as epidural stimulation systems or osteosynthesis material. The study was approved by the Ethics Committee of the City of Vienna (EK 11-124-0711). All subjects signed written informed consent prior to their participation.

**Table 1:** Neurological status of the participants with spinal cord injury according to the International Standards for Neurological Classification of spinal cord injury.

Subj.nr.	Sex	Age	Neurol. level of SCI	Years post-SCI	AIS grade	LEMS total (max. 50)	PP sensory subscore, L1–S2 (max. 28)	LT sensory subscore, L1–S2 (max. 28)	LE MAS sum score (max. 96)	Anti-spasticity medication (daily dosage)
1	F	18	T7	1	A	0	0	0	33.5	none
2	M	37	C7	16	A	0	0	0	41.5	12 mg tizanidine, 50 mg tetrazepam
3	M	20	T6	5	C	7	0	14	30	none
4	M	21	C6	2	C	8	14	28	37	50 mg baclofen
5	M	26	C4	9	C	21	14	14	37.5	50 mg baclofen, 24 mg tizanidine, 100 mg tetrazepam
6	M	61	T4	43	D	28	0	0	22	none
7	M	47	C4	4	D	38	14	14	8	none
8	M	52	C7	2	D	39	18	18	39	30 mg baclofen
9	F	53	C7	8	D	40	14	14	15.5	none
10	M	66	T4	7	D	42	14	14	17.5	10 mg baclofen

AIS, American Spinal Injury Association Impairment Scale; LEMS, lower extremity motor score; LT, light touch; LE MAS, lower extremity Modified Ashworth Scale; PP, pin prick

### Data acquisition

Surface-electromyographic (EMG) recordings were acquired from RF, BF, TA, and soleus of both legs using pairs of silver-silver chloride electrodes (Intec Medizintechnik GmbH, Klagenfurt, Austria), placed with an inter-electrode distance of 3 cm in accordance with the Surface Electromyography for the Non-Invasive Assessment of Muscles (SENIAM) recommendations (www.seniam.org). A common ground electrode was placed over the fibular head of the right leg. Abrasive paste (Nuprep, Weaver and Company, Aurora, CO) was used for skin preparation to reduce EMG electrode resistance below 5 kΩ. EMG signals were acquired using the Phoenix multichannel EMG system (EMS-Handels GmbH, Korneuburg, Austria) set to a gain of 502 (or 229, in case of amplifier saturation by large evoked potentials) over a bandwidth of 10–1000 Hz and digitized at 2048 samples per second and channel. EMG data were additionally bandpass-filtered offline between 10 and 1000 Hz using a 2^nd^ order Butterworth filter (Matlab 2017b, The MathWorks, Inc., Natick, MA). All recordings were conducted with the participants lying comfortably in the supine position.

### Stimulation procedures

The soleus-H reflex was elicited by monopolar stimulation of the posterior tibial nerve of the right leg using self-adhesive hydrogel surface electrodes (Schwamedico GmbH, Ehringshausen, Germany), with the cathode (ø 3.2 cm) in the popliteal fossa and the anode (5 x 9 cm) over the anterior aspect of the knee. A current-controlled stimulator (Stimulette r2x+, Dr. Schuhfried Medizintechnik GmbH, Moedling, Austria) was set to deliver charge-balanced, quasi-monophasic rectangular pulses of 1-ms width. The cathode position was adjusted to produce an H reflex with lowest stimulation amplitude and an isolated ankle plantar-flexion movement with increasing stimulation. The maximum H reflexes (H_max_) as well as the maximum M waves (M_max_) were determined. For the study of the H-reflex recovery cycle, the stimulation amplitude was set to produce control-H reflexes (i.e., responses to the first stimulation pulse of a pair) with a target peak-to-peak amplitude corresponding to 25% of M_max_ on the ascending limb of the recruitment curve [67,68].

PRM reflexes were evoked by means of transcutaneous lumbar SCS through a self-adhesive hydrogel surface electrode (5 x 9 cm) placed longitudinally over the spine covering the T11 and T12 spinous processes, and a pair of interconnected larger electrodes (each 8 x 13 cm) on the lower abdomen [2,26]. For transcutaneous SCS, the Stimulette r2x+ was set to deliver charge-balanced, symmetric, biphasic rectangular pulses of 1-ms width per phase. With reference to the abdominal electrodes, the paravertebral electrode pair was the anode for the first and the cathode for the second phase of the biphasic pulses (see supplementary figure in [4]). The stimulation amplitude used for studying the PRM-reflex recovery cycle was adjusted to elicit control-PRM reflexes in the right soleus with peak-to-peak amplitudes that best matched those of the control-H reflexes and, if possible, to concomitantly elicit PRM reflexes in RF, BF, TA bilaterally, as well as in the left soleus.

Recovery cycles of the H reflex (H reflex conditioned by preceding tibial nerve stimulation) and the PRM reflexes of RF, BF, TA, and soleus (PRM reflexes conditioned by preceding SCS) were studied using paired pulses with conditioning-test intervals of 20, 40, 60, 80, 100, 120, 150, 200, 250, 300, 500, 1000, 2000, and 5000 ms, always applied in this sequence. For each conditioning-test interval examined, ten paired stimuli were applied in a row, with 15 s between pairs. Continuous EMG was monitored to ensure that the lower-extremity muscles were relaxed when stimuli were applied. In case of the H reflex, the stability of the concomitantly evoked small-amplitude M wave was additionally monitored throughout the experiments to ensure constant stimulation conditions. M-wave amplitudes across conditioning-test intervals did not differ in the neurologically intact group (repeated measures analysis of variance (ANOVA), F(13,117) = .917, p = .540, ${ŋ}_{p}^{2}$ = .133) nor in the SCI group (F(13,117) = 1.526, p = .118, ${ŋ}_{p}^{2}$ = .145). Further, the M wave amplitude normalized to M_max_ varied within a range of 0.82 ± 0.16% in the neurologically intact participants and 1.63 ± 0.63% in the individuals with SCI.

### Data analysis and statistics

Data were analyzed offline using Matlab 2017b (The MathWorks, Inc., Natick, MA) and IBM SPSS Statistics 24.0 for Windows (IBM Corporation, Armonk, NY, USA). Assumptions of normality were tested using Shapiro-Wilk test and equality of variances using Levene's test. α-errors of P < .05 were considered significant. Descriptive statistics are reported as mean ± SE.

The peak-to-peak amplitudes of the H reflexes and the PRM reflexes of RF, BF, TA, and soleus of both legs acquired during the paired-stimulation paradigms were calculated. Mean peak-to-peak amplitudes ± SE were established for the group of neurologically intact individuals as well as for the group of individuals with SCI and compared using independent Student’s t-tests or Mann-Whitney U tests in case of non-normally distributed data. Effect sizes of the Student’s t-tests and the Mann-Whitney U tests were reported by Cohen’s d. The same procedure was done for the H_max_ to M_max_ ratios.

The peak-to-peak amplitudes of the reflex responses to the second stimulus of a pair were normalized to the respective first ones. For each subject group, mean ratios ± SE were obtained per conditioning-test interval for the H reflexes and the PRM reflexes of the right soleus as well as for the PRM reflexes of bilateral RF, BF, TA, and soleus (with values derived from both legs pooled). Separate two-way mixed ANOVAs were run to investigate the effect of neurological status (neurologically intact, SCI) and conditioning-test interval (repeated measures) on the recovery cycles of the H reflex and the PRM reflex of the right soleus, as well as the PRM reflexes of RF, BF, TA, and soleus considering both legs. Additionally, two-way repeated measures ANOVAs were calculated per subject group for the effect of reflex type (H reflex and PRM reflex of the right soleus) and conditioning-test interval. The recovery cycles of the PRM reflexes of the different muscles studied were compared within each subject group by calculating separate generalized linear mixed models with Satterthwaite correction for the effect of muscle and conditioning-test interval (repeated measures), to adjust for the missing PRM reflexes in RF in two individuals of each group. Subjects were modeled as hierarchical random effects. Covariance type was determined based on minimal values obtained for the Akaike Information Criterion. Assumptions of sphericity were tested with Mauchly’s test, and if voided Greenhouse-Geisser correction was applied. Effect sizes were reported by the partial eta-squared (${ŋ}_{p}^{2}$). All post-hoc tests were Bonferroni corrected.

## Results

In the neurologically intact individuals, M_max_ and H_max_ in the right soleus attained mean peak-to-peak amplitudes ± SE of 8.549 ± 1.866 mV and 2.354 ± 0.469 mV, respectively, with a mean H_max_/M_max_ of 0.307 ± 0.044. The values obtained in the individuals with SCI were, M_max_, 8.447 ± 1.437 mV, H_max_, 5.233 ± 1.405 mV, and H_max_/M_max_, 0.535 ± 0.081. No statistical differences between the groups were found for M_max_ (t_18_ = .043, P = .966, d = .019) and H_max_ (t_10.981_ = 1.944, P = .078, d = .869). H_max_/M_max_ was significantly larger in the individuals with SCI (mean difference: 0.228 ± 0.092; t_13.823_ = 2.471, P = .027, d = 1.105). The group results of the average peak-to-peak amplitudes of the control-H reflexes were 1.577 ± 0.317 mV (20.8 ± 2.1% of M_max_) in the neurologically intact individuals and 2.573 ± 0.485 mV (29.6 ± 2.7% of M_max_) in the individuals with SCI. The control-PRM reflexes of the right soleus had amplitudes of 1.668 ± 0.305 mV (21.9 ± 1.8% of M_max_) and 2.472 ± 0.409 mV (29.9 ± 2.2% of M_max_), respectively. Within subject groups, there were no differences between the control-H reflex and the control-PRM reflex nor between the ratios of the control-H reflex to M_max_ and of the control-PRM reflex to M_max_ (paired Student’s t-test; neurologically intact individuals: t_9_ = .840, P = .423, d = .266 and t_9_ = .934, P = .375, d = .295; individuals with SCI: t_9_ = .767, P = .463, d = .243 and t_9_ = .153, P = .882, d = .048).

Notably, in two of the neurologically intact individuals, H_max_ attained amplitudes corresponding to only 10.0% and 13.4% of M_max_. Hence, the targeted reflex size of 25% M_max_ could not be obtained and the respective H_max_ was used for the recovery cycles in these two individuals. Furthermore, in two individuals with SCI (subjects 3 and 7), transcutaneous SCS amplitudes could not be adjusted to elicit PRM reflexes in the right soleus with the targeted size while concomitantly obtaining PRM reflexes in all other muscles studied. Control-PRM reflexes in the right soleus of these two participants had amplitudes of 43.4% and 30.6% of M_max_, and the stimulation amplitude for the elicitation of the control-H reflexes was correspondingly increased to obtain intra-individually size-matched reflex types. Data of these four individuals (two neurologically intact, two with SCI) were excluded from the between-subjects comparisons (neurologically intact, SCI) of the recovery cycles of the H reflex and the PRM reflex of the right soleus. Between these two reduced groups with eight individuals each, there were no statistical differences between the ratios of the control-H reflex to M_max_ (neurologically intact: 23.1 ± 1.8%; SCI: 26.2 ± 1.0%; t_11.389_ = 1.509, P = .158, d = .755) nor between the ratios of the control-PRM reflex to M_max_ (neurologically intact: 23.8 ± 1.3%; SCI: 28.1 ± 2.0%; t_14_ = 1.786, P = .096, d = .893).

### Recovery cycles of size-matched H reflexes and PRM reflexes of soleus

**Fig 1:**
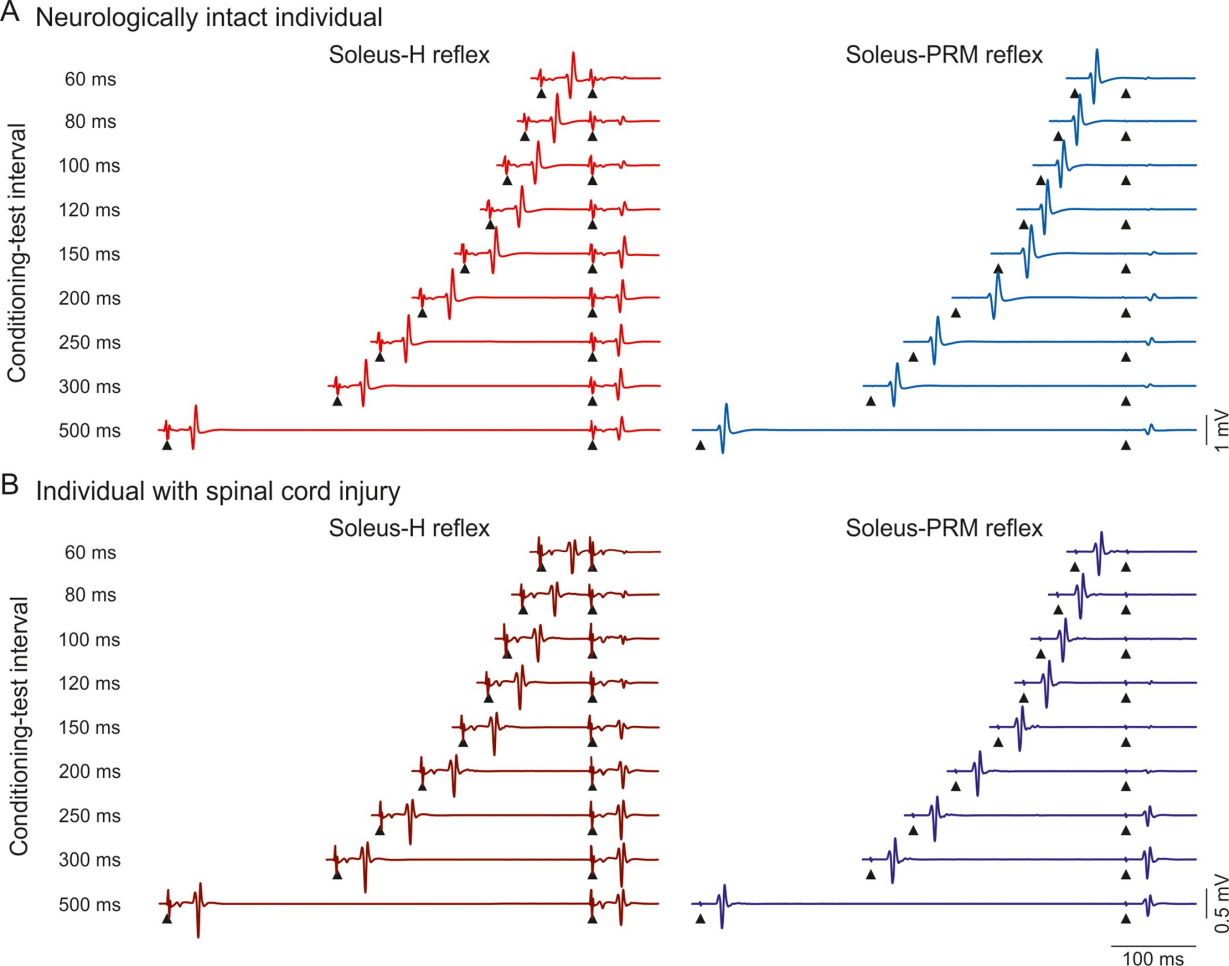
Exemplary H reflexes and PRM reflexes of soleus tested by paired-pulse paradigm. H reflexes (red EMG traces) of the right soleus and PRM reflexes (blue traces) evoked in the same muscle for a sub-set of conditioning-test intervals as indicated, derived from (A) a neurologically intact participant and (B) an individual with spinal cord injury (subject 9). Each trace is the average of ten responses. Black arrowheads mark times of stimulus application.

Representative EMG responses to paired tibial nerve stimulation and transcutaneous SCS in a neurologically intact individual and an individual with SCI are shown for a sub-set of the studied conditioning-test intervals in Fig 1.

**Fig 2:**
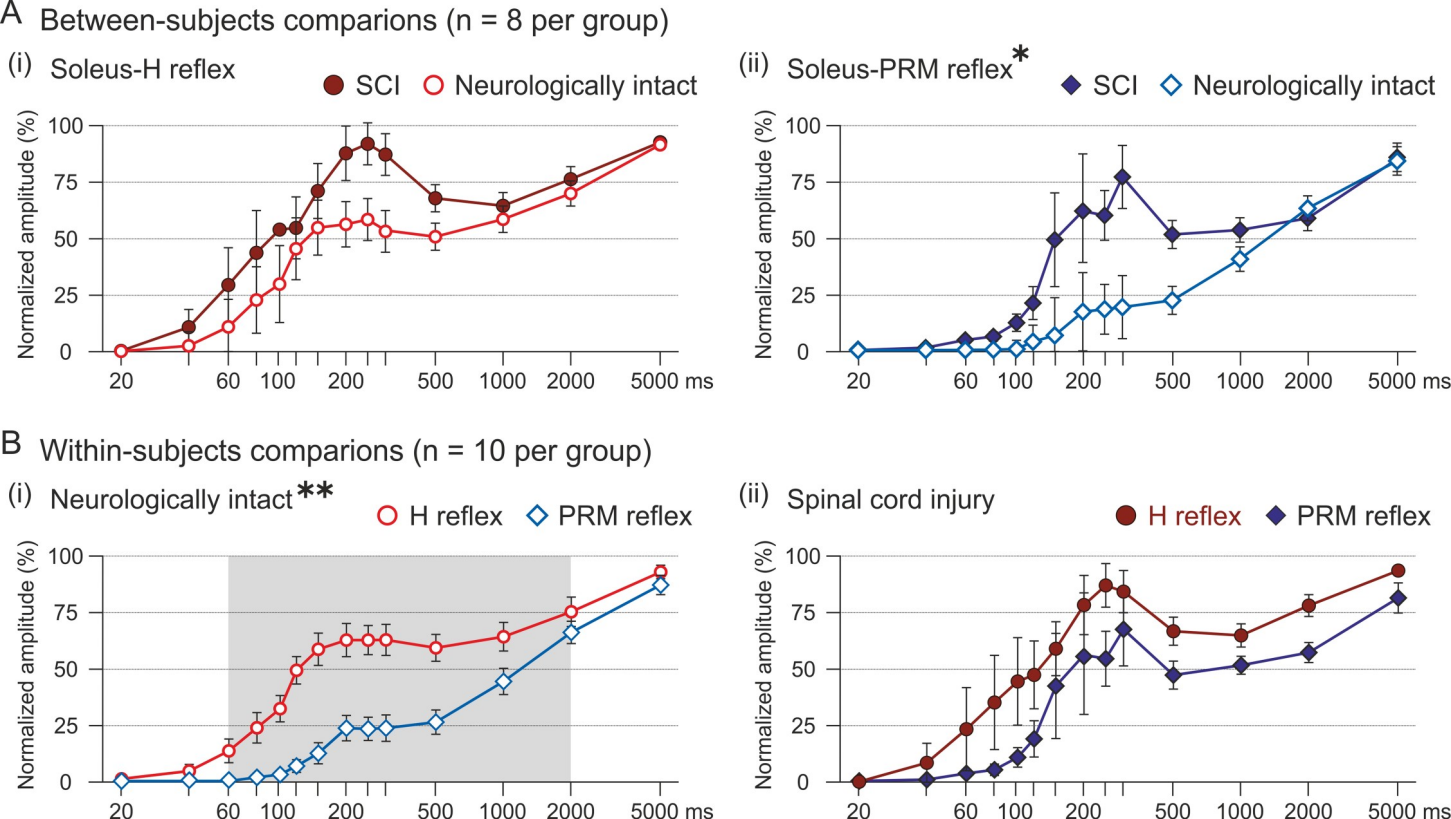
Recovery cycles of H reflexes and PRM reflexes of soleus in neurologically intact individuals and individuals with spinal cord injury. (A) Between-subjects comparison (n = 8 each) of the recovery cycles of (i) the H reflex and (ii) the PRM reflex of the right soleus with increasing conditioning-test intervals (x-axis, logarithmic scale). The y-values are group means ± SE of the normalized peak-to-peak amplitudes (second to first response) per conditioning-test interval. For the PRM reflex recovery cycle, there was a significant main effect of neurological status (*, P < .05). (B) Within-subjects comparison of recovery cycles of H reflexes and PRM reflexes (n = 10 each) in (i) neurologically intact individuals and (ii) individuals with spinal cord injury. In the neurologically intact individuals, there was a significant main effect of reflex type (**, P < .01) as well as a significant interaction between reflex type and conditioning-test interval. Shaded background marks all conditioning-test intervals with significant differences in response recovery (Bonferroni-adjusted post-hoc tests; all P < .05).

When comparing the recovery cycles of the H reflexes between subject groups (neurologically intact, SCI; n = 8 each), no statistical differences were found (Fig 2A(i)). Specifically, the mixed ANOVA of the normalized peak-to-peak amplitude (second to first response) revealed no significant effect of neurological status (F(1,14) = 2.284, P = .153, ${ŋ}_{p}^{2}$ = .140), nor a significant interaction between neurological status and conditioning-test interval (F(2.130,29.824) = .996, P = .386, ${ŋ}_{p}^{2}$ = .066). On the other hand, the PRM reflexes exhibited stronger suppression in the neurologically intact group compared to the individuals with SCI (Fig 2A(ii)). There was a significant main effect of neurological status (F(1,14) = 6.166, P = .026, ${ŋ}_{p}^{2}$ = .306). The interaction effect between neurological status and conditioning-test interval was not significant (F(1.351,18.916) = 2.484, P = .124, ${ŋ}_{p}^{2}$ = .151).

When comparing the two reflex types within the neurologically intact group (n = 10), the PRM reflexes showed a stronger suppression than the H reflexes (Fig 2B(i)). The repeated measures ANOVA demonstrated a significant main effect of reflex type (F(1,9) = 91.062, P < .0001, ${ŋ}_{p}^{2}$ = .910) as well as a significant interaction between reflex type and conditioning-test interval (F(13,117) = 11.469, P < .0001, ${ŋ}_{p}^{2}$ = .560). Bonferroni-adjusted post-hoc tests revealed significantly smaller normalized test-PRM reflexes than H reflexes at all conditioning-test intervals of 60–2000 ms (S1 Table). Within the group of individuals with SCI (Fig 2B(ii)), the recovery cycles of the H reflexes and the PRM reflexes did not differ statistically. There was no significant main effect of reflex type (F(1,9) = 4.718, P = .058, ${ŋ}_{p}^{2}$ = .344), nor a significant interaction effect between reflex type and conditioning-test interval (F(13,117) = .553, P = .886, ${ŋ}_{p}^{2}$ = .058).

### Recovery cycles of PRM reflexes of thigh and lower leg muscles

**Fig 3:**
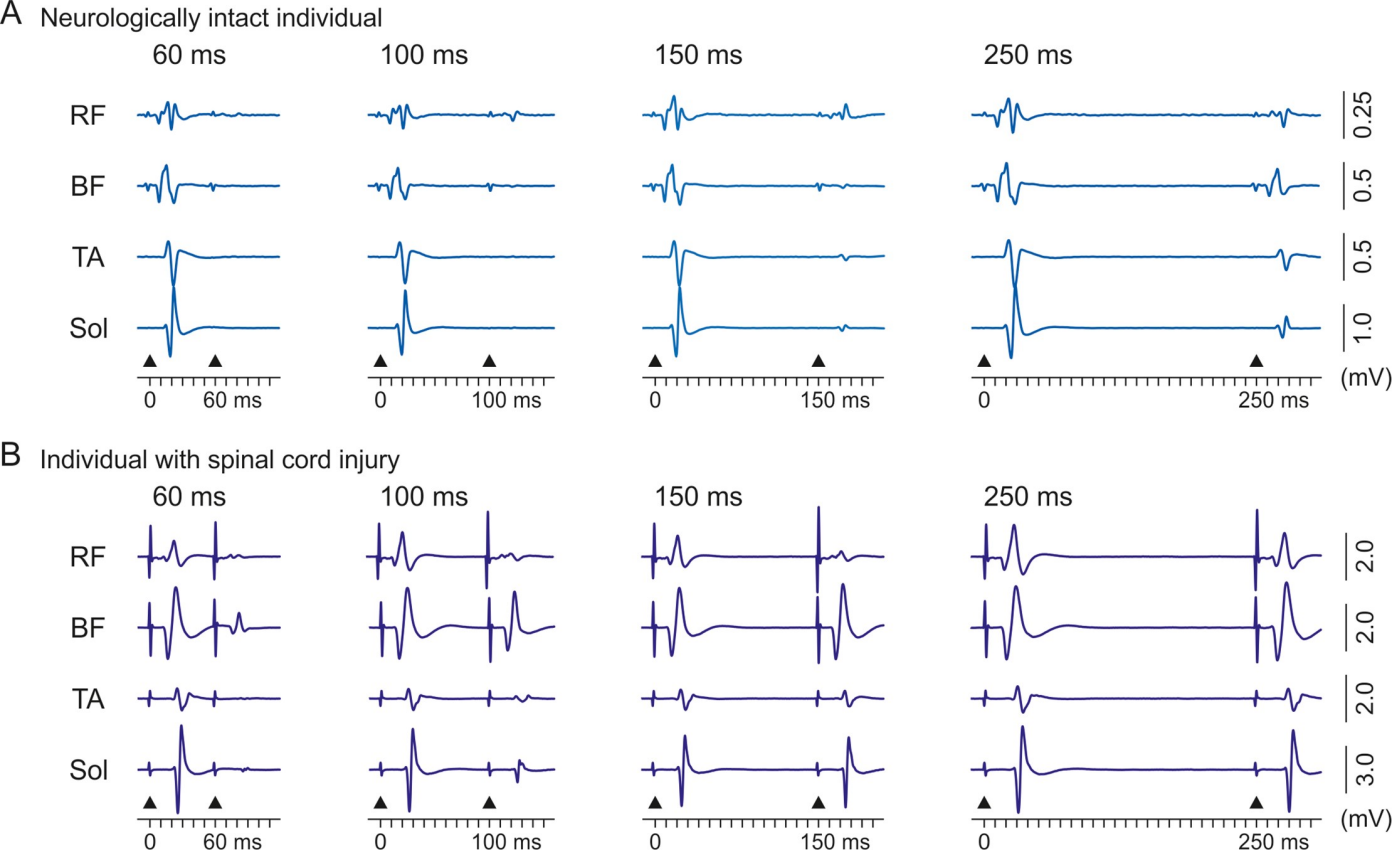
Exemplary PRM reflexes of thigh and lower leg muscles tested by paired-pulse paradigm. PRM reflexes of rectus femoris (RF), biceps femoris (BF), tibialis anterior (TA), and soleus (Sol) for a sub-set of conditioning-test intervals as indicated, derived from (A) a neurologically intact participant and (B) an individual with spinal cord injury (subject 10). Each trace is the average of ten responses. Black arrowheads mark times of stimulus application.

PRM reflexes were elicited in all participants and studied muscle groups, with the exception of responses in RF in two of the neurologically intact individuals and subjects 3 and 6 with SCI (declared as missing data). Mean peak-to-peak amplitudes ± SE of the control-PRM reflexes, considering both legs, were RF, 0.343 ± 0.201 mV in the neurologically intact individuals and 1.274 ± 0.480 mV in the individuals with SCI; BF, 1.001 ± 0.201 mV and 1.840 ± 0.448 mV; TA, 0.519 ± 0.076 mV and 0.662 ± 0.154 mV; and soleus, 1.240 ± 0.191 mV and 2.598 ± 0.460 mV. No differences in response sizes of BF (t_18_ = 1.709, P = .105, d = .540) and TA (t_13.162_ = .835, P = .419, d = .264) were detected between subject groups. The responses elicited in RF and soleus (considering left and right legs) were significantly larger in the individuals with SCI (RF, Z = 2.312, P = .02, d = .731; and soleus, t_12.001_ = 2.724, P = .018, d = .861). Representative responses to paired transcutaneous SCS for a sub-set of the studied conditioning-test intervals in a neurologically intact individual and an individual with SCI are shown in Fig 3.

**Fig 4:**
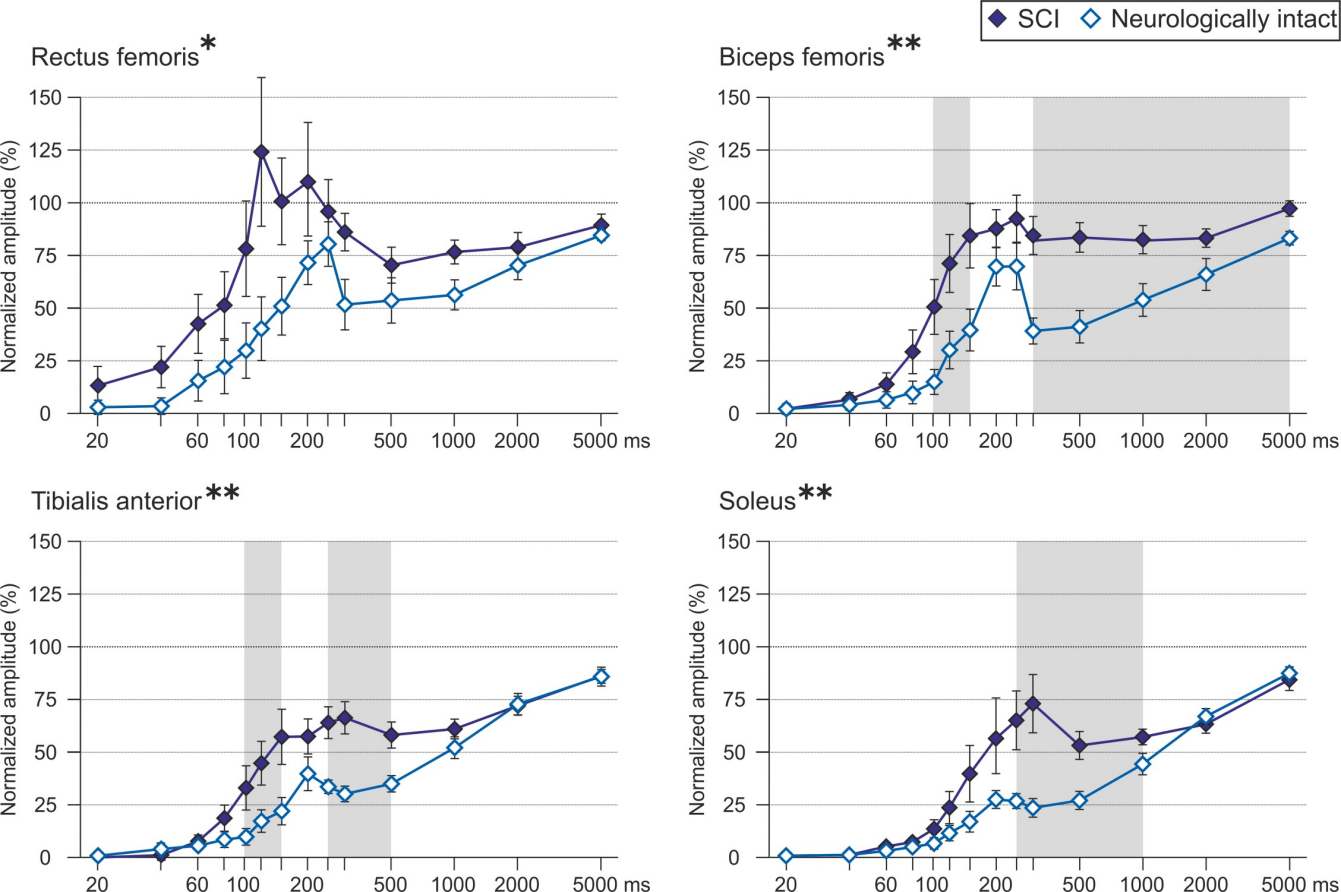
Recovery cycles of PRM reflexes of thigh and lower leg muscles in neurologically intact individuals and individuals with spinal cord injury. Recovery of PRM reflexes of rectus femoris, biceps femoris, tibialis anterior, and soleus for the neurologically intact group (light blue) and the spinal cord injured group (dark blue) with increasing conditioning-test intervals (x-axis, logarithmic scale). The y-values are group means ± SE of the normalized peak-to-peak amplitudes (second to first response) per conditioning-test interval. The PRM reflexes of all muscles studied recovered significantly faster in the individuals with spinal cord injury (significant main effects of neurological status; *, P < .05, **, P < .01). There were significant interaction effects between neurological status and conditioning-test interval in case of biceps femoris, tibialis anterior, and soleus; shaded backgrounds mark respective conditioning-test intervals with significant differences in PRM reflex recovery between subject groups (Bonferroni-adjusted post-hoc tests; all P < .05).

The recovery cycles of the PRM reflexes of RF, BF, TA and soleus of the two subject groups are compared in Fig 4. The PRM reflexes of each muscle studied exhibited stronger recovery in the individuals with SCI. The respective mixed ANOVAs revealed significant main effects of neurological status (RF, F(1,14) = 5.032, P = .042, ${ŋ}_{p}^{2}$ = .264; BF, F(1,18) = 10.145, P = .005, ${ŋ}_{p}^{2}$ = .360; TA, F(1,18) = 10.920, P = .004, ${ŋ}_{p}^{2}$ = .378; and soleus, F(1,18) = 439.337, P < .0001, ${ŋ}_{p}^{2}$ = .142). For RF, there was no significant interaction between neurological status and conditioning-test interval (F(3.062,42.871) = 1.985, P = .129, ${ŋ}_{p}^{2}$ = .264). The interaction effect was significant for BF (F(3.557,64.026) = 3.625, P = .013, ${ŋ}_{p}^{2}$ = .168), TA (F(2.969,53.449) = 4.308, P = .009, ${ŋ}_{p}^{2}$ = .193), and soleus (F(1.849,33.290) = 4.777, P = .017, ${ŋ}_{p}^{2}$ = .210). Significant results of the Bonferroni-adjusted post-hoc tests, i.e., all conditioning-test intervals exhibiting statistical differences in PRM reflex recovery between subject groups, are highlighted by shaded backgrounds in Fig 4 and specified in S2 Table.

Within the neurologically intact group, the mixed generalized linear model analysis of the PRM-reflex recovery cycles of the four muscles studied was significant (F(7,19) = 352.748, P < .0001, ${ŋ}_{p}^{2}$ = .992) (S1A(i) Fig). There was a significant main effect of muscle (F(3,15) = 8.021, P = .002, ${ŋ}_{p}^{2}$ = .616) as well as a significant interaction effect between muscle and conditioning-test interval (F(3,28) = 3.430, P = .030, ${ŋ}_{p}^{2}$ = .269). Bonferroni-adjusted pairwise post-hoc tests revealed faster and stronger recovery of PRM reflexes of the thigh than the lower leg muscles at several conditioning-test intervals between 150–500 ms (S1B(i) Fig, S3 Table).

Within the group of individuals with SCI, the mixed generalized linear model analysis of the PRM-reflex recovery cycles of the four muscles was also significant (F(7,42) = 34.205, P < .0001, ${ŋ}_{p}^{2}$ = .851), with a significant main effect of muscle (F(3,265) = 23.448, P < .0001, ${ŋ}_{p}^{2}$ = .210) as well as a significant interaction between muscle and conditioning-test interval (F(3,46) = 4.774, P = .006, ${ŋ}_{p}^{2}$ = .237) (S1A(ii) Fig). Bonferroni-adjusted pairwise post-hoc tests revealed faster and stronger recovery of PRM reflexes of the thigh than the lower leg muscles, for RF at conditioning-test intervals between 40–150 ms and for BF at 500–2000 ms (S1B(ii) Fig, S3 Table). The conditioned RF-PRM reflexes could attain even higher peak-to-peak amplitudes than the control reflexes at intervals of 120–200 ms.

## Discussion

We studied the recovery cycles of H reflexes and PRM reflexes in neurologically intact participants as well as individuals with SCI by paired stimuli at conditioning-test intervals of 20–5000 ms. When comparing the two subject groups, no statistical difference was found between the recovery cycles of the size-matched soleus-H reflexes, while the recovery of the soleus-PRM reflexes with increasing conditioning-test intervals demonstrated less depression in the individuals with SCI than in the neurologically intact participants. Within the neurologically intact group, the PRM reflex showed a stronger depression than the H reflex, whereas the recovery of the two reflex types did not differ in the SCI group. Further, between subject groups, the recovery of the PRM reflexes was stronger in bilateral RF, BF, TA, and soleus and occurred at shorter conditioning-test intervals in BF, TA, and soleus in the SCI individuals. Within the neurologically intact individuals, the PRM reflexes of the thigh muscles recovered more strongly than those of the lower leg muscles at conditioning-test intervals of 150–500 ms. In the individuals with SCI, the RF-PRM reflexes exhibited the strongest recovery of all muscles studied at conditioning-test intervals of 40–150 ms, and the conditioned response could even attain higher amplitudes than the controls. Additionally, the BF-PRM reflexes in this subject group showed stronger recovery than those of the lower leg muscles at intervals of 500–2000 ms.

### H-reflex recovery cycles do not differ between neurologically intact and SCI individuals

The H-reflex recovery cycle obtained here in the neurologically intact group was consistent with that previously reported in literature [37,69,70]. The distinct phases of inhibition of the recovery cycle with the intermediate, superimposed facilitation result from various mechanisms each of which dominates at different conditioning-test intervals. At intervals up to 100 ms, the prevailing effects include excitability changes of the Ia afferents following the conditioning stimulus [71,72] as well as afterhyperpolarization and recurrent inhibition of the activated motoneurons [54,73]. For several hundreds of milliseconds, but less than a second, presynaptic inhibition of soleus Ia afferent terminals following the conditioning stimulus reduces the transmission through the reflex pathway [48,69]. The longest-lasting mechanism with durations of up to 15 s is homosynaptic depression, i.e., a decreased probability of neurotransmitter release from the same Ia afferents that had been involved in the conditioning input [45,46,74,75]. The facilitation at around 250 ms was ascribed to two potential mechanisms; ascending activity following the conditioning stimulus to supraspinal centers that could in turn alter the excitability of spinal motoneuron pools via descending projections [76–78], or facilitation through reafferent inflow to the spinal cord caused by the relaxation (stretch) following the conditioning muscle contraction [37,70]. The subsequent reduced excitability at around 500 ms was associated with the abrupt withdrawal of Ia inflow related to the rebound shortening of the ankle extensors [70] and the activity of secondary muscle spindle afferents with inhibitory effects on the spinal motoneuron pools of extensors [79].

The H-reflex recovery cycle established for the SCI group showed no statistical differences for the tested period of 20–5000 ms when compared with the neurologically intact participants. This finding contradicted our hypothesis, which based on the common understanding that presynaptic inhibition as well as homosynaptic depression are reduced in patients with spasticity following SCI [51]. Spinal spasticity affecting the lower extremities was present in all SCI participants of our study as reflected by the clinical grading of their hypertonia based on the MAS [58] as well as their significantly increased H_max_ to M_max_ ratios [54,80]. The reason that previous studies had found decreased depression of the H reflex in individuals with spasticity could be due to methodological differences. First, most studies measured H-reflex depression following a passive Achilles-tendon stretch [47] or in the course of trains of electrical stimulation applied to the tibial nerve [49,81]. In the latter studies, stronger H-reflex recovery in SCI was demonstrated as reduced low-frequency depression, i.e., the gradual fall in H-reflex size when series of H reflexes were elicited at frequencies of 1–10 Hz was less expressed [49]. It should be noted that the depression of the H reflex is incomplete after the initial two pulses of a stimulation train [69,82]. The mechanisms underlying homosynaptic depression might be related to neurotransmitter kinetics [83,84] or short-term homosynaptic plasticity (*cf*. [50]), processes that would not be fully manifested after merely two stimuli [75], and differences between neurologically intact and SCI individuals could be more strongly expressed with trains of stimuli. Secondly, those studies that employed the paired-pulse paradigm for studying the H-reflex recovery cycles had not matched the sizes of the (unconditioned) H reflexes between neurologically intact individuals and individuals with spinal spasticity. Grey and colleagues found reduced reflex depression in spastic individuals with SCI, but, with respect to the M_max_, the control-H reflexes were more than twice as large as those in the neurologically intact participants [52]. With less than 10% of M_max_, the control H reflexes in the healthy individuals were particularly small and the conditioned H reflexes were depressed to about 40% and 60% of the controls at 2 s and 5 s, respectively, vs. 75.3% and 92.9% in the current study. Their finding might hence be related to the fact that small H reflexes exhibit stronger depression when tested by the paired-pulse paradigm [70,85].

### Dissimilarities between the soleus-PRM reflex and the H reflex

Differences in the way that the two reflexes are evoked may determine partially different spinal mechanisms underlying the respective recovery cycles. There is substantial data to suggest that PRM reflexes are essentially initiated within large-diameter afferent fibers originating from muscle spindle primary endings in the lower extremities [2–4,26,86–88]. However, due to the anatomical organization of the posterior roots at the stimulation site [6,89] and the relatively wide-spread effects of transcutaneous SCS [3,88], each single pulse at intensities as applied here simultaneously stimulated afferent fibers from various lower extremity nerves, opening the potential for heteronymous influences upon the soleus motoneuron pool.

The soleus motoneuron pool receives heteronymous monosynaptic Ia excitation from several lower extremity nerves, particularly from the femoral nerve [53,54,90–92]. Due to the sufficient duration of the homonymous monosynaptic excitatory postsynaptic potential, subliminally depolarized soleus motoneurons could be raised to firing threshold via heteronymous monosynaptic inputs [90]. A size-matched soleus PRM reflex would then require a smaller number of electrically stimulated afferents coming from the tibial nerve than the H reflex, but would depend on heteronymous facilitation.

For a duration of up to a second, the activation of multiple roots by transcutaneous SCS would produce a larger amount of presynaptic inhibition upon a successive soleus-PRM reflex than tibial nerve stimulation would exert upon the H reflex. Specifically, the simultaneous stimulation of Ia afferents from TA [69,90,93] and BF [44,94,95] by the first pulse would concomitantly exert presynaptic inhibition upon the homonymous Ia afferent terminals mediating the soleus PRM reflex evoked by the second pulse. Indeed, while heteronymous presynaptic inhibition of soleus Ia afferents was generally demonstrated by a conditioning tendon tap or brief tendon vibration (3 shocks at 200 Hz), it can be evoked by single-pulse electrical stimulation as well [44,96,97].

A further consequence of the multi-source presynaptic inhibition evoked by the first pulse would be a suppression of heteronymous Ia facilitation of a succeeding soleus-PRM reflex [54,90]. Finally, the heteronymous monosynaptic Ia facilitation adding to the size of the soleus-PRM reflex would, by itself, be subject to homosynaptic depression [56].

### Soleus-PRM reflex depression is stronger in neurologically intact than in SCI individuals

As opposed to the H-reflex recovery cycles, the recovery cycles of the soleus-PRM reflexes were significantly different between the subject groups, with a striking suppression of the PRM reflexes in the intact group for durations characteristic for presynaptic inhibition. This observation supports the assumption of a multi-source heteronymous presynaptic inhibition of the soleus-PRM reflex and suggests its dramatic decrease after SCI [50]. In the SCI group, the long-lasting heteronymous presynaptic inhibition from flexor nerves following the first pulse would have been weaker [95] and heteronymous facilitation of the conditioned soleus PRM reflex evoked by the second pulse (due to reduced presynaptic inhibition of monosynaptic heteronymous Ia facilitation) would have been stronger [48]. Further, reduced homosynaptic depression of heteronymous facilitation from multiple roots [56] contributing to the PRM reflex size could have accentuated the reduced PRM reflex suppression in the SCI group.

### H-reflex and soleus-PRM reflex recovery cycles differ in neurologically intact but not in SCI individuals

The significantly stronger suppression of the soleus-PRM reflex in the intact group at conditioning-test intervals of 60–2000 ms complementarily strengthens the hypothesis of its dependence on additional facilitatory inputs (which are suppressed with repeated stimulation) when compared with the H reflex, and/or that the multi-root input produces stronger cumulative inhibition. The lack of difference between the H-reflex and soleus-PRM reflex recovery cycles in the SCI group could consequently be explained by dysfunction of the additional inhibitory mechanisms recruited by the multi-root stimulation.

### PRM-reflex recovery cycles of thigh and lower leg muscles are different between neurologically intact and SCI individuals

The recovery of the PRM reflexes was stronger in all studied muscles and faster in BF, TA, and soleus in the SCI individuals. At the intensities applied here, concomitantly evoking PRM reflexes in thigh and lower leg muscles, the PRM reflexes of each tested muscle would receive cumulative homonymous and heteronymous group I presynaptic inhibition from the multiple posterior roots stimulated [54], which contain afferent fibers of all major lower-extremity nerves. Among the known heteronymous actions, quadriceps receives strong presynaptic inhibition from knee and ankle flexor nerves, BF from TA, and soleus largely from TA and the hamstrings muscle group [44]. Heteronymous presynaptic inhibition from TA suppresses soleus- and quadriceps-H reflexes as well as reduces the heteronymous Ia facilitation from the femoral nerve to the soleus-H reflex [90]. Heteronymous presynaptic inhibition from BF depresses the soleus- and TA-H reflexes and the amount of femoral nerve-induced heteronymous Ia facilitation of the soleus- and TA-H reflexes [94]. Thus, heteronymous presynaptic inhibition would affect both homonymous monosynaptic Ia activation as well as the heteronymous monosynaptic Ia facilitation, upon which the PRM reflexes of each of the muscles studied likely depend [54,92,98]. The collective deficient presynaptic inhibition in the individuals with SCI would then explain the stronger and faster PRM reflex recovery for the different muscles tested when compared to the intact group.

At this point it is noteworthy to recall that regarding the H reflex, the recovery cycle is reflex-size dependent, and small H reflexes exhibit a slow recovery [39,70]. Here, the absolute EMG peak-to-peak amplitudes of RF- and soleus-PRM reflexes (considering left and right legs) were significantly smaller in the individuals with intact nervous system. However, the recovery cycles of the soleus-PRM reflex had shown a similar discrepancy between the subject groups (intact vs. SCI) even when the reflex sizes were size-matched (right legs only). Hence, any potential influence of reflex size on the recovery cycles was likely limited to the RF-PRM reflexes.

### PRM-reflex recovery cycles differ between thigh and lower leg muscles

In both subject groups, the PRM reflexes of RF and BF showed a stronger recovery than the lower leg muscles. The stronger recovery could be a not yet described motor control feature inherent to the stretch reflexes of the thigh muscle groups given by a lesser degree of homosynaptic depression and/or homonymous presynaptic inhibition upon repetitive activation compared with the lower leg muscles. Independently, the discrepancy between the recovery cycles of the thigh vs. the lower leg PRM reflexes was specifically apparent at intervals corresponding to the intermediate reflex recovery at 200 ms and 250 ms. This may suggest that reafferent inflow to the spinal cord following the muscle contraction generated by the first pulse [37,70] might play a more prominent role in the thigh muscles. Consistent with this assumption is also the sudden drop of the thigh muscle recovery cycles at 300 ms, that could be explained by the withdrawal of Ia inflow caused by the subsiding stretch or rebound shortening of the homonymous muscle [70].

## Conclusions

The soleus-PRM reflex and the H reflex differ in one of their key characteristics, i.e., their recovery cycles when tested by paired pulses, in neurologically intact individuals. The marked suppression of the soleus-PRM reflex could be explained by its dependence on heteronymous Ia facilitation as well as a strong cumulative heteronymous presynaptic inhibition following the multi-root stimulation. Such difference between the reflex types will have important implications when PRM reflexes are used in motor control studies, as their excitability changes caused by conditioning inputs may be influenced by presynaptic modulation of their homonymous and heteronymous connections. The substantial difference of the soleus-PRM reflex recovery cycles between intact and SCI individuals could be used as a sensitive measure in interventional studies of spasticity, as it likely results from the collective dysfunction of a wide range of spinal inhibitory mechanisms following SCI, probed simultaneously by transcutaneous SCS. The recruitment of multiple sources of inhibition acting at a presynaptic site might be also a key mechanism underlying the antispasticity effect of SCS [99,100]. The different recovery behavior of thigh and lower leg muscle reflexes may imply that during movement, presynaptic spinal mechanisms would maintain different levels of efficacy of the Ia-motoneuron synapse for diverse muscles.

## Supporting information

S1 Fig Within-subject comparison of the recovery cycles of PRM reflexes of thigh and lower leg muscles. (A) Recovery cycles of PRM reflexes of rectus femoris, biceps femoris, tibialis anterior, and soleus for (i) the neurologically intact group and (ii) individuals with spinal cord injury with increasing conditioning-test intervals (x-axis, logarithmic scale). The y-values are group means ± SE of the normalized peak-to-peak amplitudes (second to first response) per conditioning-test interval. Same recovery cycles as displayed in Fig 4, but reorganized to facilitate within-subject comparison and error bars omitted for clarity. For both subject groups, there were significant main effects of muscle and significant muscle x conditioning-test interval interactions (**, P < .01). Details of significant results of the Bonferroni-adjusted post-hoc tests are given in B (all P < .05) as well as S3 Table.
(TIF)

S1 Table Mean normalized peak-to-peak amplitudes ± SE of the H reflex and PRM reflex of soleus, respectively, derived from the neurologically intact individuals (n = 10) at conditioning-intervals exhibiting significant differences between reflex types along with p-values of Bonferroni-adjusted post-hoc pairwise comparisons. (PDF)

S2 Table Mean normalized peak-to-peak amplitudes ± SE of PRM reflexes of biceps femoris, tibialis anterior and soleus, respectively, at conditioning-intervals exhibiting significant differences between subject groups along with p-values of Bonferroni-adjusted post-hoc pairwise comparisons. (PDF)

S3 Table Mean normalized peak-to-peak amplitudes ± SE of PRM reflexes of rectus femoris (RF), biceps femoris (BF), tibialis anterior (TA) and soleus, respectively, derived from neurologically intact participants and individuals with spinal cord injury at conditioning-test intervals exhibiting significant differences between muscles along with p-values of Bonferroni-adjusted post-hoc pairwise comparisons. (PDF)

## References

[1] Minassian K, Jilge B, Rattay F, Pinter MM, Binder H, Gerstenbrand F, et al Stepping-like movements in humans with complete spinal cord injury induced by epidural stimulation of the lumbar cord: electromyographic study of compound muscle action potentials. Spinal Cord. 2004;42: 401–416. 10.1038/sj.sc.3101615 15124000

[2] Minassian K, Persy I, Rattay F, Dimitrijevic MR, Hofer C, Kern H. Posterior root-muscle reflexes elicited by transcutaneous stimulation of the human lumbosacral cord. Muscle Nerve. 2007;35: 327–336. 10.1002/mus.20700 17117411

[3] Ladenbauer J, Minassian K, Hofstoetter US, Dimitrijevic MR, Rattay F. Stimulation of the human lumbar spinal cord with implanted and surface electrodes: A computer simulation study. IEEE Trans Neural Syst Rehabil Eng. 2010;18: 637–645. 10.1109/TNSRE.2010.2054112 21138794

[4] Hofstoetter US, Freundl B, Binder H, Minassian K. Common neural structures activated by epidural and transcutaneous lumbar spinal cord stimulation: Elicitation of posterior root-muscle reflexes. PLoS One. 2018;13: e0192013 10.1371/journal.pone.0192013 29381748 PMC5790266

[5] Rattay F, Minassian K, Dimitrijevic MR. Epidural electrical stimulation of posterior structures of the human lumbosacral cord: 2. quantitative analysis by computer modeling. Spinal Cord. 2000;38: 473–489. 10.1038/sj.sc.3101039 10962608

[6] Minassian K, Persy I, Rattay F, Pinter MM, Kern H, Dimitrijevic MR. Human lumbar cord circuitries can be activated by extrinsic tonic input to generate locomotor-like activity. Hum Mov Sci. 2007;26: 275–295. 10.1016/j.humov.2007.01.005 17343947

[7] Capogrosso M, Wenger N, Raspopovic S, Musienko P, Beauparlant J, Bassi Luciani L, et al A computational model for epidural electrical stimulation of spinal sensorimotor circuits. J Neurosci. 2013;33: 19326–19340. 10.1523/JNEUROSCI.1688-13.2013 24305828 PMC6618777

[8] Formento E, Minassian K, Wagner F, Mignardot JB, Le Goff-Mignardot CG, Rowald A, et al Electrical spinal cord stimulation must preserve proprioception to enable locomotion in humans with spinal cord injury. Nat Neurosci. 2018;21: 1728–1741. 10.1038/s41593-018-0262-6 30382196 PMC6268129

[9] Courtine G, Harkema SJ, Dy CJ, Gerasimenko YP, Dyhre-Poulsen P. Modulation of multisegmental monosynaptic responses in a variety of leg muscles during walking and running in humans. J Physiol. 2007;582: 1125–1139. 10.1113/jphysiol.2007.128447 17446226 PMC2075265

[10] Sayenko DG, Angeli C, Harkema SJ, Edgerton VR, Gerasimenko YP. Neuromodulation of evoked muscle potentials induced by epidural spinal-cord stimulation in paralyzed individuals. J Neurophysiol. 2014;111: 1088–1099. 10.1152/jn.00489.2013 24335213 PMC3949232

[11] Angeli CA, Boakye M, Morton RA, Vogt J, Benton K, Chen Y, et al Recovery of Over-Ground Walking after Chronic Motor Complete Spinal Cord Injury. N Engl J Med. 2018;379: 1244–1250. 10.1056/NEJMoa1803588 30247091

[12] Gill ML, Grahn PJ, Calvert JS, Linde MB, Lavrov IA, Strommen JA, et al Neuromodulation of lumbosacral spinal networks enables independent stepping after complete paraplegia. Nat Med. 2018;24: 1677–1682. 10.1038/s41591-018-0175-7 30250140

[13] Wagner FB, Mignardot J-B, Le Goff-Mignardot CG, Demesmaeker R, Komi S, Capogrosso M, et al Targeted neurotechnology restores walking in humans with spinal cord injury. Nature. 2018;563: 65–71. 10.1038/s41586-018-0649-2 30382197

[14] Calvert JS, Grahn PJ, Strommen JA, Lavrov IA, Beck LA, Gill ML, et al Electrophysiological Guidance of Epidural Electrode Array Implantation over the Human Lumbosacral Spinal Cord to Enable Motor Function after Chronic Paralysis. J Neurotrauma. 2019;36: 1451–1460. 10.1089/neu.2018.5921 30430902 PMC6482916

[15] Murg M, Binder H, Dimitrijevic MR. Epidural electric stimulation of posterior structures of the human lumbar spinal cord: 1. muscle twitches—a functional method to define the site of stimulation. Spinal Cord. 2000;38: 394–402. 10.1038/sj.sc.3101038 10962598

[16] Minassian K, Hofstoetter US, Dzeladini F, Guertin PA, Ijspeert A. The Human Central Pattern Generator for Locomotion: Does It Exist and Contribute to Walking? Neuroscientist. 2017;23: 649–663. 10.1177/1073858417699790 28351197

[17] Hofstoetter US, Krenn M, Danner SM, Hofer C, Kern H, McKay WB, Mayr W, Minassian K. Augmentation of Voluntary Locomotor Activity by Transcutaneous Spinal Cord Stimulation in Motor-Incomplete Spinal Cord-Injured Individuals. Artif Organs. 2015;39:E176–186. 10.1111/aor.12615 26450344

[18] Minassian K, Hofstoetter US, Danner SM, Mayr W, Bruce JA, McKay WB, et al Spinal rhythm generation by step-induced feedback and transcutaneous posterior root stimulation in complete spinal cord-injured individuals. Neurorehabil Neural Repair. 2016;30:233–243. 10.1177/1545968315591706 26089308

[19] Gad P, Gerasimenko Y, Zdunowski S, Turner A, Sayenko D, Lu DC, et al Weight Bearing Over-ground Stepping in an Exoskeleton with Non-invasive Spinal Cord Neuromodulation after Motor Complete Paraplegia. Front Neurosci. 2017;11:333 10.3389/fnins.2017.00333 28642680 PMC5462970

[20] Sayenko DG, Rath M, Ferguson AR, Burdick JW, Havton LA, Edgerton VR, et al Self-Assisted Standing Enabled by Non-Invasive Spinal Stimulation after Spinal Cord Injury. J Neurotrauma. 2019;36: 1435–1450. 10.1089/neu.2018.5956 30362876 PMC6482915

[21] Hofstoetter U, Freundl B, Danner S, Krenn M, Mayr W, Binder H, et al Transcutaneous spinal cord stimulation induces temporary attenuation of spasticity in individuals with spinal cord injury. J Neurotrauma. 2019 8 9 10.1089/neu.2019.6588 31333064

[22] Hofstoetter US, Minassian K, Hofer C, Mayr W, Rattay F, Dimitrijevic MR. Modification of reflex responses to lumbar posterior root stimulation by motor tasks in healthy subjects. Artif Organs. 2008;32: 644–648. 10.1111/j.1525-1594.2008.00616.x 18782137

[23] Saito A, Masugi Y, Nakagawa K, Obata H, Nakazawa K. Repeatability of spinal reflexes of lower limb muscles evoked by transcutaneous spinal cord stimulation. Tremblay F, editor. PLoS One. 2019;14: e0214818 10.1371/journal.pone.0214818 30947310 PMC6448839

[24] Milosevic M, Masugi Y, Obata H, Sasaki A, Popovic MR, Nakazawa K. Short-term inhibition of spinal reflexes in multiple lower limb muscles after neuromuscular electrical stimulation of ankle plantar flexors. Exp Brain Res. 2019;237: 467–476. 10.1007/s00221-018-5437-6 30460394

[25] Andrews JC, Stein RB, Roy FD. Reduced postactivation depression of soleus H reflex and root evoked potential after transcranial magnetic stimulation. J Neurophysiol. 2015;114: 485–492. 10.1152/jn.01007.2014 25995355 PMC4509403

[26] Minassian K, Hofstoetter US, Rattay F. Transcutaneous lumbar posterior root stimulation for motor control studies and modification of motor activity after spinal cord injury In: Dimitrijevic M, Kakulas B, McKay W, Vrbova G, editors. Restorative neurology of spinal cord injury. New York: Oxford University Press; 2011 pp. 226–255.

[27] Hoffmann P. Beiträge zur Kenntnis der menschlichen Reflexe mit besonderer Berücksichtigung der elektrischen Erscheinungen. Arch Anat Physiol. 1910;1: 223–246.

[28] Hoffmann P. Über die Beziehungen der Sehnenreflexe zur willkürlichen Bewegung und zum Tonus. Z Biol. 1918;68: 351–370.

[29] Magladery JW, McDougal DB. Electrophysiological studies of nerve and reflex activity in normal man. I. Identification of certain reflexes in the electromyogram and the conduction velocity of peripheral nerve fibers. Bull Johns Hopkins Hosp. 1950;86: 265–290. 15414383

[30] Burke D, Gandevia SC, McKeon B. The afferent volleys responsible for spinal proprioceptive reflexes in man. J Physiol. 1983;339: 535–552. 10.1113/jphysiol.1983.sp014732 6887033 PMC1199177

[31] Burke D, Gandevia SC, McKeon B. Monosynaptic and oligosynaptic contributions to human ankle jerk and H-reflex. J Neurophysiol. 1984;52: 435–448. 10.1152/jn.1984.52.3.435 6090608

[32] Magladery JW, Porter WE, Park AM, Teasdall RD. Electrophysiological studies of nerve and reflex activity in normal man. IV. The two-neurone reflex and identification of certain action potentials from spinal roots and cord. Bull Johns Hopkins Hosp. 1951;88: 499–519. 14839348

[33] Katz R. Presynaptic inhibition in humans: a comparison between normal and spastic patients. J Physiol Paris. 1999;93: 379–385. 10.1016/s0928-4257(00)80065-0 10574126

[34] Dy CJ, Gerasimenko YP, Edgerton VR, Dyhre-Poulsen P, Courtine G, Harkema SJ. Phase-dependent modulation of percutaneously elicited multisegmental muscle responses after spinal cord injury. J Neurophysiol. 2010;103: 2808–2820. 10.1152/jn.00316.2009 20357075 PMC2867577

[35] Hoffmann P. Untersuchungen über die refraktäre Periode des menschlichen Rückenmarks. Z Biol. 1924;81: 37–48.

[36] Schenck E. [Studies on the silent period following a bineuronal (proprioreceptive) reflex in man]. Pflugers Arch Gesamte Physiol Menschen Tiere. 1951;253: 286–300. 10.1007/bf00363395 14833863

[37] Paillard J. Réflexes et Régulations d’Origine Proprioceptive chez l’Homme Etude Neurophysiologique et Psychophysiologique. Paris: Librairie Arnette; 1955.

[38] Magladery J, Teasdall R, Park A, Porter W. Electrophysiological studies of nerve and reflex activity in normal man. V. Excitation and inhibition of two-neurone reflexes by afferent impulses in the same trunk. Bull Johns Hopkins Hosp. 1951;88: 520–537. 14839349

[39] Olsen PZ, Diamantopoulos E. Excitability of spinal motor neurones in normal subjects and patients with spasticity, Parkinsonian rigidity, and cerebellar hypotonia. J Neurol Neurosurg Psychiatry. 1967;30: 325–331. 10.1136/jnnp.30.4.325 6055341 PMC496193

[40] Schieppati M. The Hoffmann reflex: a means of assessing spinal reflex excitability and its descending control in man. Prog Neurobiol. 1987;28: 345–376. 10.1016/0301-0082(87)90007-4 3588965

[41] Minassian K, Hofstoetter U, Rattay F, Mayr W, Dimitirjevic M. Posterior root-muscle reflexes and the H reflex in humans: Electrophysiological comparison Neuroscience Meeting Planner, Chicago, IL: Society for Neuroscience (online) 2009. Program No. 658.12.

[42] Andrews JC, Stein RB, Roy FD. Post-activation depression in the human soleus muscle using peripheral nerve and transcutaneous spinal stimulation. Neurosci Lett. 2015;589: 144–149. 10.1016/j.neulet.2015.01.041 25600855

[43] Eccles JC, Schmidt RF, Willis WD. Presynaptic inhibition of the spinal monosynaptic reflex pathway. J Physiol. 1962;161: 282–297. 10.1113/jphysiol.1962.sp006886 13889059 PMC1359623

[44] Iles JF, Roberts RC. Inhibition of monosynaptic reflexes in the human lower limb. J Physiol. 1987;385: 69–87. 10.1113/jphysiol.1987.sp016484 2958622 PMC1192337

[45] Crone C, Nielsen J. Methodological implications of the post activation depression of the soleus H-reflex in man. Exp Brain Res. 1989;78: 28–32. 10.1007/bf00230683 2591515

[46] Hultborn H, Illert M, Nielsen J, Paul A, Ballegaard M, Wiese H. On the mechanism of the post-activation depression of the H-reflex in human subjects. Exp Brain Res. 1996;108: 450–462. 10.1007/bf00227268 8801125

[47] Nielsen J, Petersen N, Ballegaard M, Biering-Sørensen F, Kiehn O. H-reflexes are less depressed following muscle stretch in spastic spinal cord injured patients than in healthy subjects. Exp Brain Res. 1993;97: 173–176. 10.1007/bf00228827 8131827

[48] Faist M, Mazevet D, Dietz V, Pierrot-Deseilligny E. A quantitative assessment of presynaptic inhibition of Ia afferents in spastics. Differences in hemiplegics and paraplegics. Brain A J Neurol. 1994;117: 1449–1455. 10.1093/brain/117.6.1449 7820579

[49] Schindler-Ivens S, Shields RK. Low frequency depression of H-reflexes in humans with acute and chronic spinal-cord injury. Exp Brain Res. 2000;133: 233–241. 10.1007/s002210000377 10968224 PMC4034370

[50] Nielsen J, Willerslev-Olsen M, Lorentzen J. Pathophysiology of Spasticity In: Pandyan A, Hermens H, Conway B, editors. Neurological Rehabilitation Spasticity and Contractures in Clinical Practice and Research. Boca Raton: Imprint CRC Press; 2018 pp. 25–57.

[51] Elbasiouny SM, Moroz D, Bakr MM, Mushahwar VK. Management of spasticity after spinal cord injury: current techniques and future directions. Neurorehabil Neural Repair. 2010;24: 23–33. 10.1177/1545968309343213 19723923 PMC2860542

[52] Grey MJ, Klinge K, Crone C, Lorentzen J, Biering-Sørensen F, Ravnborg M, et al Post-activation depression of soleus stretch reflexes in healthy and spastic humans. Exp Brain Res. 2008;185: 189–197. 10.1007/s00221-007-1142-6 17932663

[53] Bergmans J, Delwaide PJ, Gadea-Ciria M. Short-latency effects of low-threshold muscular afferent fibers on different motoneuronal pools of the lower limb in man. Exp Neurol. 1978;60: 380–385. 10.1016/0014-4886(78)90091-2 658210

[54] Pierrot-Deseilligny E, Burke D. The Circuitry of the Human Spinal Cord. Cambridge: Cambridge University Press; 2012.

[55] Delwaide PJ, Cordonnier M, Charlier M. Functional relationships between myotatic reflex arcs of the lower limb in man: investigation by excitability curves. J Neurol Neurosurg Psychiatry. 1976;39: 545–554. 10.1136/jnnp.39.6.545 950566 PMC492346

[56] Lamy JC, Wargon I, Baret M, Ben Smail D, Milani P, Raoul S, et al Post-activation depression in various group I spinal pathways in humans. Exp Brain Res. 2005;166: 248–262. 10.1007/s00221-005-2360-4 16078020

[57] Kirshblum S, Waring W. Updates for the International Standards for Neurological Classification of Spinal Cord Injury. Phys Med Rehabil Clin N Am. 2014;25: 505–517. 10.1016/j.pmr.2014.04.001 25064785

[58] Bohannon RW, Smith MB. Interrater reliability of a modified Ashworth scale of muscle spasticity. Phys Ther. 1987;67: 206–207. 10.1093/ptj/67.2.206 3809245

[59] Hulme A, MacLennan WJ, Ritchie RT, John VA, Shotton PA. Baclofen in the elderly stroke patient its side-effects and pharmacokinetics. Eur J Clin Pharmacol. 1985;29: 467–469. 10.1007/bf00613463 3912190

[60] Tse FL, Jaffe JM, Bhuta S. Pharmacokinetics of orally administered tizanidine in healthy volunteers. Fundam Clin Pharmacol. 1987;1: 479–488. 10.1111/j.1472-8206.1987.tb00581.x 3447935

[61] Mathias CJ, Luckitt J, Desai P, Baker H, el Masri W, Frankel HL. Pharmacodynamics and pharmacokinetics of the oral antispastic agent tizanidine in patients with spinal cord injury. J Rehabil Res Dev. 1989;26: 9–16. 2600869

[62] Shellenberger MK, Groves L, Shah J, Novack GD. A controlled pharmacokinetic evaluation of tizanidine and baclofen at steady state. Drug Metab Dispos. 1999;27: 201–204. 9929503

[63] Malanga G, Reiter RD, Garay E. Update on tizanidine for muscle spasticity and emerging indications. Expert Opin Pharmacother. 2008;9: 2209–2215. 10.1517/14656566.9.12.2209 18671474

[64] Baumgärtner MG, Cautreels W, Langenbahn H. Biotransformation and pharmacokinetics of tetrazepam in man. Arzneimittelforschung. 1984;34: 724–729. 6148954

[65] Bun H, Philip F, Berger Y, Necciari J, Al-Mallah NR, Serradimign A, et al Plasma levels and pharmacokinetics of single and multiple dose of tetrazepam in healthy volunteers. Arzneimittelforschung. 1987;37: 199–202. 2883980

[66] Delwaide PJ, Pennisi G. Tizanidine and electrophysiologic analysis of spinal control mechanisms in humans with spasticity. Neurology. 1994;44: S21–27. 7970007

[67] Pierrot-Deseilligny E, Mazevet D. The monosynaptic reflex: a tool to investigate motor control in humans. Interest and limits. Clin Neurophysiol. 2000;30: 67–80. 10.1016/s0987-7053(00)00062-9 10812576

[68] Knikou M. The H-reflex as a probe: pathways and pitfalls. J Neurosci Methods. 2008;171: 1–12. 10.1016/j.jneumeth.2008.02.012 18394711

[69] Kohn AF, Floeter MK, Hallett M. Presynaptic inhibition compared with homosynaptic depression as an explanation for soleus H-reflex depression in humans. Exp Brain Res. 1997;116: 375–380. 10.1007/pl00005765 9348136

[70] Kagamihara Y, Hayashi A, Okuma Y, Nagaoka M, Nakajima Y, Tanaka R. Reassessment of H-reflex recovery curve using the double stimulation procedure. Muscle Nerve. 1998;21: 352–360. 10.1002/(sici)1097-4598(199803)21:3<352::aid-mus9>3.0.co;2-9 9486864

[71] Morita H, Shindo M, Yanagawa S, Yanagisawa N. Neuromuscular response in man to repetitive nerve stimulation. Muscle Nerve. 1993;16: 648–654. 10.1002/mus.880160611 8389002

[72] Kiernan MC, Mogyoros I, Burke D. Differences in the recovery of excitability in sensory and motor axons of human median nerve. Brain A J Neurol. 1996;119: 1099–1105. 10.1093/brain/119.4.1099 8813274

[73] Eccles JC, Fatt P, Koketsu K. Cholinergic and inhibitory synapses in a pathway from motor-axon collaterals to motoneurones. J Physiol. 1954;126: 524–562. 10.1113/jphysiol.1954.sp005226 13222354 PMC1365877

[74] Beswick F, Evanson J. Homosynaptic depression of the monosynaptic reflex following its activation. J Physiol. 1957;135: 400–411. 10.1113/jphysiol.1957.sp005719 13406749 PMC1358843

[75] Kuno M. Mechanism of facilitation and depression of the excitatory synaptic potential in spinal motoneurons. J Physiol. 1964;175: 100–112. 10.1113/jphysiol.1964.sp007505 14241151 PMC1357087

[76] Shimamura M, Mori S, Matsushima S, Fujimori B. On the spino-bulbo-spinal reflex in dogs, monkeys and man. Jpn J Physiol. 1964;14: 411–421. 10.2170/jjphysiol.14.411 14200821

[77] Taborikova H, Provini L, Decandia M. Evidence that muscle stretch evokes long-loop reflexes from higher centres. Brain Res. 1966;2: 192–194. 10.1016/0006-8993(66)90026-6 5968923

[78] Taborikova H, Sax DS. Conditioning of H-reflexes by a preceding subthreshold H-reflex stimulus. Brain A J Neurol. 1969;92: 203–212. 10.1093/brain/92.1.203 5774028

[79] Bianconi R, Granit R, Reis DJ. The effect of extensor muscle spindles and tendon organs on homonymous motoneurones in relation to gamma-bias and curarization. Acta Physiol Scand. 1964;61: 331–347. 14209252

[80] Nakazawa K, Kawashima N, Akai M. Enhanced stretch reflex excitability of the soleus muscle in persons with incomplete rather than complete chronic spinal cord injury. Arch Phys Med Rehabil. 2006;87: 71–75. 10.1016/j.apmr.2005.08.122 16401441

[81] Calancie B, Broton JG, Klose KJ, Traad M, Difini J, Ayyar DR. Evidence that alterations in presynaptic inhibition contribute to segmental hypo- and hyperexcitability after spinal cord injury in man. Electroencephalogr Clin Neurophysiol. 1993;89: 177–186. 10.1016/0168-5597(93)90131-8 7686850

[82] Ishikawa K, Ott K, Porter RW, Stuart D. Low frequency depression of the H wave in normal and spinal man. Exp Neurol. 1966;15: 140–156. 10.1016/0014-4886(66)90039-2 5934660

[83] Cisi RRL, Kohn AF. H-reflex depression simulated by a biologically realistic motoneuron network. Conf Proc IEEE Eng Med Biol Soc. 2007;2007: 2713–2716. 10.1109/IEMBS.2007.4352889 18002555

[84] Kohn AF, Floeter MK, Hallett M. A model-based approach for the quantification of H reflex depression in humans. Conf Proc IEEE Eng Med Biol Soc. 1995;1995: 1233–1234.

[85] Diamantopoulos E, Zander Olsen P. Excitability of motor neurones in spinal shock in man. J Neurol Neurosurg Psychiatry. 1967;30: 427–431. 10.1136/jnnp.30.5.427 6062993 PMC496219

[86] Zhu Y, Starr A, Haldeman S, Chu JK, Sugerman RA. Soleus H-reflex to S1 nerve root stimulation. Electroencephalogr Clin Neurophysiol. 1998;109: 10–14. 10.1016/s0924-980x(97)00058-1 11003059

[87] Kitano K, Koceja DM. Spinal reflex in human lower leg muscles evoked by transcutaneous spinal cord stimulation. J Neurosci Methods. 2009;180: 111–115. 10.1016/j.jneumeth.2009.03.006 19427537

[88] Danner SM, Hofstoetter US, Ladenbauer J, Rattay F, Minassian K. Can the Human Lumbar Posterior Columns Be Stimulated by Transcutaneous Spinal Cord Stimulation? A Modeling Study. Artif Organs. 2011;35: 257–262. 10.1111/j.1525-1594.2011.01213.x 21401670 PMC4217151

[89] Lang J, Geisel U. [Lumbosacral part of the dural sac and the topography of its contents]. Morphol Med. 1983;3: 27–46. 6877253

[90] Hultborn H, Meunier S, Morin C, Pierrot-Deseilligny E. Assessing changes in presynaptic inhibition of I a fibres: a study in man and the cat. J Physiol. 1987;389: 729–756. 10.1113/jphysiol.1987.sp016680 3681741 PMC1192104

[91] Meunier S, Penicaud A, Pierrot-Deseilligny E, Rossi A. Monosynaptic Ia excitation and recurrent inhibition from quadriceps to ankle flexors and extensors in man. J Physiol. 1990;423: 661–675. 10.1113/jphysiol.1990.sp018046 2388162 PMC1189781

[92] Meunier S, Pierrot-Deseilligny E, Simonetta M. Pattern of monosynaptic heteronymous Ia connections in the human lower limb. Exp Brain Res. 1993;96: 534–544. 10.1007/bf00234121 8299754

[93] Morin C, Pierrot-Deseilligny E, Hultborn H. Evidence for presynaptic inhibition of muscle spindle Ia afferents in man. Neurosci Lett. 1984;44: 137–142. 10.1016/0304-3940(84)90071-5 6231494

[94] Nielsen J, Petersen N. Is presynaptic inhibition distributed to corticospinal fibres in man? J Physiol. 1994;477: 47–58. 10.1113/jphysiol.1994.sp020170 8071888 PMC1155573

[95] Nielsen J, Petersen N, Crone C. Changes in transmission across synapses of Ia afferents in spastic patients. Brain A J Neurol. 1995;118: 995–1004. 10.1093/brain/118.4.995 7655894

[96] Mizuno Y, Tanaka R, Yanagisawa N. Reciprocal group I inhibition on triceps surae motoneurons in man. J Neurophysiol. 1971;34: 1010–1017. 10.1152/jn.1971.34.6.1010 4329961

[97] Morita H, Crone C, Christenhuis D, Petersen NT, Nielsen JB. Modulation of presynaptic inhibition and disynaptic reciprocal Ia inhibition during voluntary movement in spasticity. Brain A J Neurol. 2001;124: 826–837. 10.1093/brain/124.4.826 11287381

[98] Mao CC, Ashby P, Wang M, McCrea D. Synaptic connections from large muscle afferents to the motoneurons of various leg muscles in man. Exp Brain Res. 1984;56: 341–350. 10.1007/bf00236290 6090196

[99] Hofstoetter US, McKay WB, Tansey KE, Mayr W, Kern H, Minassian K. Modification of spasticity by transcutaneous spinal cord stimulation in individuals with incomplete spinal cord injury. J Spinal Cord Med. 2014;37: 202–211. 10.1179/2045772313Y.0000000149 24090290 PMC4066429

[100] Estes SP, Iddings JA, Field-Fote EC. Priming Neural Circuits to Modulate Spinal Reflex Excitability. Front Neurol. 2017;8: 17 10.3389/fneur.2017.00017 28217104 PMC5289977

