# Supplementary figures and images for: Recovery cycles of posterior root-muscle reflexes evoked by transcutaneous spinal cord stimulation and of the H reflex in individuals with intact and injured spinal cord
Source: PLoS One. 2019 Dec 26;14(12):e0227057. doi: 10.1371/journal.pone.0227057 (PMC6932776; doi:10.1371/journal.pone.0227057)

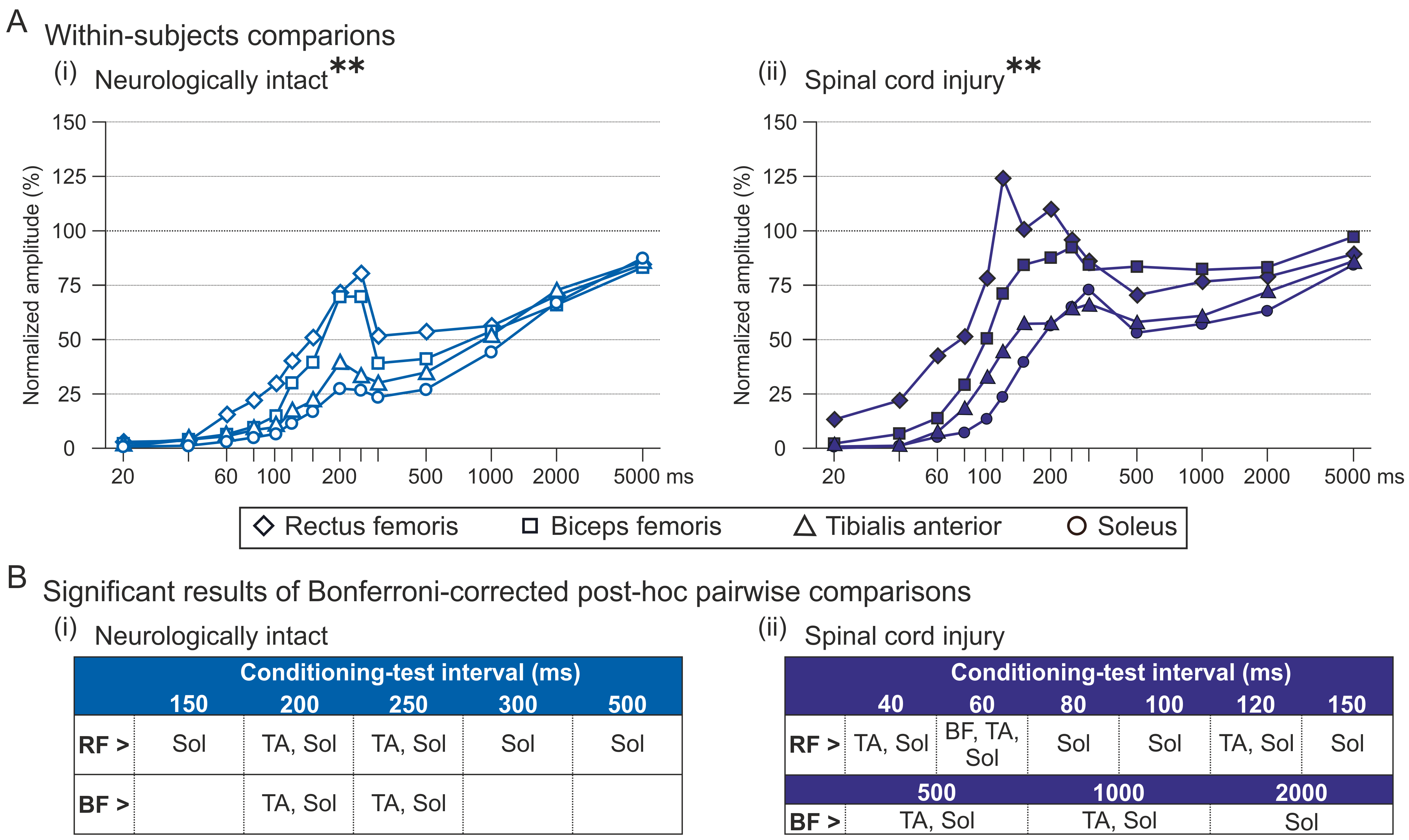

Supplement: S1 Fig — (A) Recovery cycles of PRM reflexes of rectus femoris, biceps femoris, tibialis anterior, and soleus for (i) the neurologically intact group and (ii) individuals with spinal cord injury with increasing conditioning-test intervals (x-axis, logarithmic scale). The y-values are group means ± SE of the normalized peak-to-peak amplitudes (second to first response) per conditioning-test interval. Same recovery cycles as displayed in Fig 4, but reorganized to facilitate within-subject comparison and error bars omitted for clarity. For both subject groups, there were significant main effects of muscle and significant muscle x conditioning-test interval interactions (**, P < .01). Details of significant results of the Bonferroni-adjusted post-hoc tests are given in B (all P < .05) as well as S3 Table. (TIF) [file pone.0227057.s001.tif]
