# Supplementary material for: Recovery cycles of posterior root-muscle reflexes evoked by transcutaneous spinal cord stimulation and of the H reflex in individuals with intact and injured spinal cord
Source: PLoS One. 2019 Dec 26;14(12):e0227057. doi: 10.1371/journal.pone.0227057 (PMC6932776; doi:10.1371/journal.pone.0227057)
Supplement: S1 Table — (PDF) [file pone.0227057.s002.pdf]

S1 Table. Mean normalized peak-to-peak amplitudes (± SE) of the H reflex and PRM reflex of soleus, respectively, derived from the neurologically intact individuals (n = 10) at conditioning-intervals exhibiting significant differences between reflex types along with p-values of Bonferroni-adjusted post-hoc pairwise comparisons.

Conditioning-test interval	Soleus-H reflex	Soleus-PRM reflex	p-value
60 ms	0.134 ± 0.051	0.004 ± 0.003	.026
80 ms	0.237 ± 0.065	0.015 ± 0.008	.009
100 ms	0.322 ± 0.057	0.028 ± 0.015	.001
120 ms	0.493 ± 0.059	0.066 ± 0.026	< .0001
150 ms	0.586 ± 0.070	0.123 ± 0.045	< .0001
200 ms	0.627 ± 0.072	0.234 ± 0.054	< .0001
250 ms	0.627 ± 0.056	0.231 ± 0.050	< .0001
300 ms	0.593 ± 0.060	0.234 ± 0.057	< .0001
500 ms	0.574 ± 0.052	0.261 ± 0.052	< .0001
1000 ms	0.642 ± 0.055	0.441 ± 0.056	< .0001
2000 ms	0.753 ± 0.057	0.659 ± 0.048	.020
