# Supplementary material for: Recovery cycles of posterior root-muscle reflexes evoked by transcutaneous spinal cord stimulation and of the H reflex in individuals with intact and injured spinal cord
Source: PLoS One. 2019 Dec 26;14(12):e0227057. doi: 10.1371/journal.pone.0227057 (PMC6932776; doi:10.1371/journal.pone.0227057)
Supplement: S2 Table — (PDF) [file pone.0227057.s003.pdf]

S2 Table. Mean normalized peak-to-peak amplitudes (± SE) of PRM reflexes of biceps femoris, tibialis anterior and soleus, respectively, at conditioning-intervals exhibiting significant differences between subject groups along with p-values of Bonferroni-adjusted post-hoc pairwise comparisons.

Conditioning-test interval	Neurologically intact	Spinal cord injury	p-value
**Biceps femoris**			
100 ms	0.140 ± 0.056	0.506 ± 0.127	.017
120 ms	0.293 ± 0.087	0.711 ± 0.134	.017
150 ms	0.388 ± 0.096	0.842 ± 0.150	.020
300 ms	0.383 ± 0.058	0.844 ± 0.087	< .0001
500 ms	0.403 ± 0.074	0.835 ± 0.066	< .0001
1000 ms	0.531 ± 0.075	0.825 ± 0.063	.008
2000 ms	0.652 ± 0.073	0.831 ± 0.040	.045
5000 ms	0.826 ± 0.029	0.972 ± 0.033	.004
**Tibialis anterior**			
100 ms	0.090 ± 0.036	0.328 ± 0.103	.043
120 ms	0.166 ± 0.051	0.447 ± 0.102	.024
150 ms	0.214 ± 0.062	0.573 ± 0.129	.022
250 ms	0.331 ± 0.029	0.641 ± 0.073	.001
300 ms	0.297 ± 0.034	0.664 ± 0.074	< .0001
500 ms	0.346 ± 0.036	0.582 ±0.059	.003
**Soleus**			
250 ms	0.263 ± 0.032	0.652 ± 0.138	.013
300 ms	0.230 ± 0.041	0.732 ± 0.136	.002
500 ms	0.265 ± 0.040	0.532 ± 0.063	.002
1000 ms	0.440 ± 0.048	0.572 ± 0.034	.038
