# Supplementary material for: Recovery cycles of posterior root-muscle reflexes evoked by transcutaneous spinal cord stimulation and of the H reflex in individuals with intact and injured spinal cord
Source: PLoS One. 2019 Dec 26;14(12):e0227057. doi: 10.1371/journal.pone.0227057 (PMC6932776; doi:10.1371/journal.pone.0227057)
Supplement: S3 Table — (PDF) [file pone.0227057.s004.pdf]

S3 Table. Mean normalized peak-to-peak amplitudes (± SE) of PRM reflexes of rectus femoris (RF), biceps femoris (BF), tibialis anterior (TA) and soleus, respectively, derived from neurologically intact participants and individuals with spinal cord injury at conditioning-test intervals exhibiting significant differences between muscles along with p-values of Bonferroni-adjusted post-hoc pairwise comparisons.

Conditioning-test interval	PRM reflex				p-value
**Neurologically intact**					
150 ms	RF	0.509 ± 0.134	soleus	0.163 ± 0.046	.035
200 ms	RF	0.715 ± 0.101	TA	0.393 ± 0.077	.038
			soleus	0.270 ± 0.040	.002
	BF	0.690 ± 0.090	TA	0.393 ± 0.077	.040
			soleus	0.270 ± 0.040	.002
250 ms	RF	0.803 ± 0.102	TA	0.331 ± 0.029	.001
			soleus	0.263 ± 0.032	.0001
	BF	0.692 ± 0.109	TA	0.331 ± 0.029	.005
			soleus	0.263 ± 0.032	.001
300 ms	RF	0.516 ± 0.116	soleus	0.230 ± 0.041	.011
500 ms	RF	0.536 ± 0.104	soleus	0.265 ± 0.040	.025
**Spinal cord injury**					
40 ms	RF	0.221 ± 0.94	TA	0.007 ± 0.007	.003
			soleus	0.009 ± 0.007	.004
60 ms	RF	0.425 ± 0.136	BF	0.139 ± 0.050	.011
			TA	0.074 ± 0.026	.001
			soleus	0.049 ± 0.020	.001
80 ms	RF	0.513 ± 0.156	soleus	0.069 ± 0.022	.004
100 ms	RF	0.781 ± 0.224	soleus	0.133 ± 0.040	.004
120 ms	RF	1.240 ± 0.351	TA	0.447 ± 0.102	.016
			soleus	0.234 ± 0.074	.001
150 ms	RF	1.005 ± 0.203	soleus	0.396 ± 0.132	.037
500 ms	BF	0.835 ± 0.066	TA	0.581 ± 0.059	.033
			soleus	0.532 ± 0.063	.007
1000 ms	BF	0.824 ± 0.063	TA	0.611 ± 0.059	.020
			soleus	0.572 ± 0.034	.004
2000 ms	BF	0.831 ± 0.040	soleus	0.635 ± 0.047	.036
